# Putting the methodological brakes on claims to measure national happiness through Twitter: Methodological limitations in social media analytics

**DOI:** 10.1371/journal.pone.0180080

**Published:** 2017-09-07

**Authors:** Eric Allen Jensen

**Affiliations:** Department of Sociology, University of Warwick, Coventry, United Kingdom; Indiana University Bloomington, UNITED STATES

## Abstract

With the rapid global proliferation of social media, there has been growing interest in using this existing source of easily accessible ‘big data’ to develop social science knowledge. However, amidst the big data gold rush, it is important that long-established principles of good social research are not ignored. This article critically evaluates Mitchell *et al*.’s (2013) study, ‘The Geography of Happiness: Connecting Twitter Sentiment and Expression, Demographics, and Objective Characteristics of Place’, demonstrating the importance of attending to key methodological issues associated with secondary data analysis.

With the rapid global proliferation of social media, there has been growing interest in using this existing source of easily accessible data to develop social science knowledge. The extraordinarily large sample sizes that are made possible by social media-based research have made such ‘big data’ studies particularly alluring to both scientific journals and news media. However, amidst the big data gold rush, long-established principles of good social research have too often been ignored. The consequences of flouting these principles have not been fully articulated to date. This article uses a published example by Mitchell *et al*. [1] to illustrate how methodological limitations can undermine big data research. The main issues identified are [1] inferential over-extensions resulting in over-claiming [2], limitations in the operationalization of key concepts, [3] de facto sampling bias, and [4] a failure to account for the inherent shortcomings of this form of secondary data.

Big data analysts are using social media data from platforms such as Twitter to address major social research questions about populations, which have been traditionally investigated using social surveys or other methods [1]. Mitchell *et al*. describe their research focus as follows:

How does living in urban areas relate to well-being? Such an undertaking is part of a general program seeking to quantify and explain the evolving cultural character–the stories–of cities, as well as geographic places of larger and smaller scales. […] Our overall aim in this paper is to investigate how geographic place correlates with and potentially influences societal levels of happiness. (p. 1)

This study is making the inferential leap from easily observable social media behavior to unmediated (offline) social reality such as individuals’ emotional states and attitudes. Digital systems have interfaces with the non-digital world, but these two worlds are not co-extensive. In order to validate and calibrate the use of Twitter data to make claims about the non-digital world, there would need to be evidence to establish the ground truth of Twitter use and speech patterns. It cannot simply be assumed that the physical world has been “virtualized” by the transfer of actions and emotions online [2]. While affected by similar issues, online social life is distinguishable from offline social reality. Distinctive cultural patterns arise in online communities, which have their own characteristics that do not necessarily map onto the offline reality for the individuals participating in those communities [3, 4]. That is, the extent of overlap between individuals’ online and offline behavior and psychology has not been well established, but there is certainly reason to suspect that a gap exists between reported and actual behavior [5]. While some inferences about individuals based on Twitter or Facebook data are plausible, a sophisticated analytic framework would be required to limit the risk of introducing uncontrolled error into the analysis. Moreover, sociological research is needed to establish the ways in which Twitter users envisage their online contributions, the particular imagined audiences and assumed conventions they are operating with and the nature of the speech genres that delimit or guide their speech. Yet, the validation research required to establish the relationship between social media content and social reality has not yet been conducted. Some studies [1] are left in the position of making the questionable assumption that there is a 1:1 relationship between social media content and offline emotional states or attitudes.

## Face validity of operationalization

Mitchell *et al*. “measure the overall average happiness of people located in cities”[1] as follows:

Our methodology […] uses word frequency distributions collected from a large corpus of geolocated messages or ‘tweets’ posted on Twitter, with individual words scored for their happiness independently by users of Amazon’s Mechanical Turk service. (p. 1)

Thus, words were given a single score that remained static regardless of context.

A total of roughly 10,000 of these individual words have been scored by users of Amazon’s Mechanical Turk service on a scale of 1 (sad) to 9 (happy), resulting in a measure of average happiness for each given word. For example, ‘rainbow’ is one of the happiest words in the list with a score of **havg~8:1**, while ‘earthquake’ is one of the saddest, with **havg~1:9** (p. 2).

The level of quality that could be expected from this use of the Mechanical Turk service is unclear. However, even if individual words have been reliably scored for this study, the use of a simple dictionary method for categorizing the inherent ‘happiness’ of a tweet is clearly prone to a substantial amount of error (e.g. due to the use of irony, different meanings of words in different contexts, words that negate meaning such as ‘not’, etc.). To take the examples cited in this article by the authors, while ‘rainbow’ may be most commonly used in positive sentences, it is conceivable someone might say “What a terrible disappointment that season finale was: It was like finding there is no pot of gold at the end of the rainbow!”. The word ‘rainbow’ in this negative phrase would be given a very ‘happy’ score based on the study’s methods. Likewise, someone might use ‘earthquake’ in a metaphorical sense, for example, “Meeting Samantha was like an earthquake. I am in love and my entire landscape has shifted!”. The word ‘never’ is defined as sad, so an optimistic tweet saying “Never say never!” would get a doubly sad score. There are innumerable examples of this kind. Essentially, this way of ascribing sentiment to tweets using a ‘bag of words’ approach is likely to be prone to error. Indeed, this fact is widely understood within the social scientific discipline of linguistics, where issues such as word sense disambiguation [6] and linguistic compositionality have been studied for decades [7, 8].

Remarkably the authors claim the lack of precision associated with this method as a badge of objectivity:

By ignoring the context of words we gain both a computational advantage and a degree of impartiality; we do not need to decide a priori whether a given word has emotional content, thereby reducing the number of steps in the algorithm and hopefully reducing experimental bias. (p. 3)

To check for the accuracy in the categorization of tweets as ‘happy’ or ‘sad’, the likely level of error in this ‘sentiment analysis’ approach could have been quantified by having human coders blind score a random selection of tweets to see how well these scores corresponded to the automated score. However, this type of quality control check was not conducted. Instead, it is merely asserted that the happiness scores for the tweets are reliable. These sentiment scores (with their unknown levels of error) then become the basis for all the subsequent analyses.

Moreover, this kind of ‘context-free’ sentiment analysis is fundamentally at odds with social media-based communication, as well as much of the methodological work that has been conducted in the natural language processing field in computer science. People using social media can respond to ideas in unpredictable ways, drawing upon a communication backdrop that may move back and forth between online social media and offline social or professional contexts. The ways in which some people react online or offline may in turn influence others, who may respond either online or offline. This makes the online setting a hive of mutual influence in which the direct, unfiltered communication of ideas between unaffiliated and disinterested individuals is a rare scenario. Therefore, taking context into account in at least some manner is a basic requirement for valid identification of message sentiment on Twitter or anywhere else online. Natural language processing researchers account for some of the contextual issues visible online through analyses of ngrams and domain sensitive lexical resources. However, there is still a major research gap in understanding the relationship between online and offline behavior. In sum, the ‘happiness’ part of the analysis in Mitchell *et al*.’s study [1] requires a more sophisticated sentiment measure.

## The importance of a representative sample

Mitchell *et al*. analyze 10 million tweets [1], an enormous sample size to be sure. But as we know, large sample sizes do not necessarily equate to good or even accurate research. To make valid generalizations, researchers must ensure data are representative of the target population.

Mitchell *et al*. use only the US-based geotagged tweets [1] (approximately 1% of messages) from within Twitter’s ‘garden house’ feed (10% of all messages). This means that the sample was comprised of one-tenth of one percent of all Twitter messages during the 2011 calendar year. While the sample size is large in absolute terms, there is no evidence provided to indicate that this small percentage of the total number of tweets is representative of the broader population of Twitter messages and users, let alone the entire population (which is mostly comprised of non-users of Twitter). That is, there may be systematic bias in terms of who is represented in the Twitter ‘garden hose’. Indeed, it has already been demonstrated that those who geotag their tweets may be systematically different than the overall Twitter population [9]. This methodological issue has been demonstrated in the UK in two recent studies, showing a demographic gap between Twitter users and the general population [10] and furthermore between those who geotag their tweets and the general population of Twitter users [5]. In the United States, research by Pew Research Center [11] has shown demographic gaps based on age, gender, socio-economic status, ethnicity and community type (urban, rural or suburban). In addition to likely biases in terms of the demographic profile of users included in the sample, there is also the possibility that people may be more likely to turn on geotagging in certain circumstances thereby introducing a further uncontrolled source of bias.

When researchers find themselves with easily accessible data, there is a temptation to apply those data to interesting research questions and populations, even when there are limitations in the representativeness of the sample. In the present case, Mitchell *et al*. have used data based on social surveys using representative sampling of states and cities, and treated it as comparable to the geotagged Twitter data from the same states and cities. However, the demographic characteristics of Twitter users are different from the general population in a number of ways, which are only partially understood at this point. For example, there is a substantial gender bias towards men, who represent 71.8% of Twitter users according to one estimate [12]. Therefore, there is reason to believe that combining these two sources of data is problematic.

A further limitation is the very concept of ‘average happiness’, which is used in this study. Given the high likelihood of sampling bias, the presentation of ‘average happiness’ scores for all fifty states is questionable. For these average scores to be accurate the following would have to hold true:

1. Twitter content would have to provide an accurate window into individuals’ offline happiness.
2. The automated sentiment analysis tool would have to be able to accurately identify happiness and sadness in Twitter content.
3. Twitter users would have to be representative of the general population at a state level.
4. The concept of ‘average happiness’ would have to be meaningful in principle.

In fact, none of the above points have been established in published research literature, thereby casting doubt on the entire study and its claims.

## Big data analysis is secondary analysis

The challenges affecting the kind of big data analysis discussed in this article have long affected social scientists attempting to use existing data to develop new knowledge. Known in the methodological literature as ‘secondary’ analysis, there are well-understood limitations affecting such research [13, 14].

- Some individuals may have contributed more to the dataset than others. For example, prolific Twitter users will have a much greater representation in aggregated datasets like the one considered in the present review. This means prolific users are over-represented in the data.
- Some individuals may have been excluded from the sample in ways that are not fully known or understood. In the present case, those who do not use Twitter at all or who never turned on geotagging during the sample period of 2011 would be absent from the sample.
- It may be not be feasible to source information about the creator of the data. This limitation brings with it the risk of developing knowledge claims that inadvertently ignore key information about the individuals from whom data have been collected or the context within which data were collected. This certainly applies in the present case, where details about the Twitter user producing the sampled tweets are not taken into account. For example, a resident of one state, say Massachusetts, may be sending geotagged tweets in another state, say Pennsylvania. Mitchell *et al*. would have attributed this hypothetical individual’s tweets to Pennsylvania’s happiness score, but her demographic profile would be accounted for in Massachusetts. Multiplied over millions of individuals, this could introduce substantial amounts of uncontrolled error.
- Reliance on byproducts of social action rather than direct observation or questioning can result in specious interpretation. For example, the assumption Mitchell *et al*. make is that tweet content provides a reliable index of real human happiness occurring offline. This assumption is not evidenced (nor has it been demonstrated empirically to date). Thus, there is likely to be error introduced in the gap between the real human emotion (i.e. happiness) and the easily observable byproduct (i.e. the words in a tweet).
- When analyzing existing data, one has to make the best of what is available. This can result in analyses that do not account for all relevant predictor variables. This can lead to developing specious causal inferences about relationships between variables that are in fact mediated by other factors outside of the analyst’s view.

Finally, a basic precept of statistical analysis also bears repeating: Correlation is not causation. We must avoid a naïve belief in the power of large sample sizes to overcome all sources of bias or confounding variables. Of course, the limitations identified in this article do not mean that social media studies should all be dismissed. Rather, like all other social research methods, they must establish a reasonable basis for the inferences and generalizations they present. Such studies will be on much firmer ground if they seek to generalize to particular categories of social media users, rather than to the general population. Moreover, efforts to use social media data to generalize to broader offline populations would need to be underpinned by supplemental evidence in the form of surveys or other field work to show what types of sampling bias these data might be introducing.

## Conclusion

As people make their way around the web, they leave all kinds of digital traces. These forms of data, including social media, offer the real prospect of developing useful social research insights. However, this essay highlights the point that the enthusiasm for accessing and analyzing these digital traces should not outpace sound methodology. Indeed, recent work such as the Ribeiro *et al*.’s benchmarking analysis [15] shows greater attention to the reliability of different sentiment analysis tools.

Research cannot start from the assumption that speech on Twitter can be straightforwardly treated as similar to offline conversation data. Rather, it is possible that a variety of conversational strategies and practices are unique to Twitter (just as they would be unique to other social media sites that set different speech parameters). Indeed, all of the factors that affect social reality offline also play out online: power, voice, symbolic representation, identity, leadership, struggles over scarce resources and visual representations continue to exert strong influence on the web. This raises complexities that must be addressed before claims about happiness and its causes can be approached using tweets and correlations.

Vast sample sizes increase the risk of identifying specious statistically significant results. A deductive, hypothesis-driven approach is required in order to minimize the risk of identifying significant statistical relationships that merely reflect random sampling variation rather than real patterns in the population. This article argued that long-established methodological principles governing secondary analysis in the social sciences hold the keys to understanding the methodological limitations discussed. As has been argued previously [16], ‘Twitter data has serious methodological challenges that are rarely addressed by those who embrace it. When researchers approach a data set, they need to understand–and publicly account for–not only the limits of the data set, but also the limits of which questions they can ask of a data set and what interpretations are appropriate’.
